# Gender differences in “optimistic” information processing in uncertain decisions

**DOI:** 10.3758/s13415-023-01075-7

**Published:** 2023-02-23

**Authors:** Uma R. Karmarkar

**Affiliations:** Rady School of Management, School of Global Policy and Strategy, University of California, San Diego, 9500 Gilman Drive #0553, La Jolla, CA 92093-0553 USA

**Keywords:** Decision-making, Uncertainty, Ambiguity, Risk, Gender, Information processing

## Abstract

Decision-makers often are faced with uncertain situations in which they have incomplete information. While risky decisions include the probabilities of the possible outcomes, ambiguous decisions involve both unknown probabilities and unknown outcomes. Prior research has suggested that there are differences in how men and women evaluate risk, but evidence related to gender and ambiguity is mixed. The present work approaches this problem from a novel angle, focusing on the use of information that is present rather than the impact of information that is absent. It examines how individuals assign value in uncertain decisions based on the partial information they do have. While a main effect of gender on value is not observed, there is an enhanced “optimism bias” in how both favorable and unfavorable information influences the subjective value of ambiguous financial prospects for male compared to female participants. Unpacking these effects suggests multiple mechanisms, including a significant contribution of risk processing. Specifically, favorable and unfavorable information are over- and underweighted respectively in male participants’ estimated likelihood of a winning outcome, and unfavorable information is underweighted in estimating certainty. There also is an interaction of gender and risk preferences, such that value increases more for male participants as the subjectively estimated likelihood of winning increases. A second experiment demonstrates this risk interaction effect is also observed for objective probabilities of winning, suggesting that the relationship between value and risk uses similar mechanisms across layers of uncertainty.

**Supplementary Information:**

The online version contains supplementary material available at 10.3758/s13415-023-01075-7.

A wide range of consequential decisions, from choosing a restaurant to considering medical treatments or selecting financial investments involve various forms of uncertainty. In economic frameworks, such uncertainty can be characterized in “layers” of risk and ambiguity (Ellsberg, 1961; Knight, 1921). Risk reflects situations in which people have full information about the probabilities of uncertain outcomes. Under ambiguity, however, the information needed to estimate these probabilities is incomplete—either partially or entirely unknown. Although uncertain decisions are often described as “risky,” real-world settings rarely offer precise or concrete probability information about potential outcomes (see also Tymula et al., 2012). Thus, ambiguity may offer a more flexible model for subjective experiences of everyday choice that encompasses estimated (or perceived) risk as well as how certain people feel about those estimates. The uncertainty represented by ambiguity reflects separable constructs with distinguishable neural mechanisms from risk (Blankenstein et al., 2017; Blankenstein et al., 2018; Hsu et al., 2005; Huettel et al., 2006; Wu et al., 2021). Indeed, individuals generally find the absence of information in ambiguous contexts aversive in ways that extend beyond sensitivity to risk and have distinct impacts on decision-making (Camerer & Weber, 1992; Halevy, 2007; Trautmann and Van De Kuilen, 2015).

A meaningful volume of prior research has found gender differences in evaluating and taking risks across different domains such as professional choices or personal health and notably so in economic and financial decisions (Byrnes et al., 1999; Charness & Gneezy, 2012; Eagly et al., 1995; Eckel & Grossman, 2008). Overall, despite a number of moderating contextual factors, men appear to be less sensitive to risk than women, investing and committing more in risky prospects. Several studies have expanded on these effects to better understand gender differences in competitive professional settings as well as financial markets (Dwyer et al., 2002; Faccio et al., 2016; Sunden & Surette, 1998) However, the research findings exploring gender differences related to ambiguity are more complex than those for risk.

Powell and Ansic (1997) used an insurance choice paradigm to show that women were more risk averse but not necessarily ambiguity averse when making decisions in the domain of loss. This contrasts with a study exploring the domain of gains, involved offering teenaged (aged 15-16 years) participants a series of ambiguous gambles in which they needed to guess the color of a ball drawn from an urn of ten balls (Borghans et al., 2009). The “degree” of the gambles’ ambiguity was varied by changing the precision of the color information. For example, participants could find that five balls were blue and five yellow (no ambiguity) or that two to eight balls were blue and two to eight balls were yellow (moderately high ambiguity). Women had lower reservation prices across the range of gambles, suggesting a higher sensitivity to uncertainty that could be attributed to risk. However, controlling for that main effect, men showed more sensitivity to ambiguity (Borghans et al., 2009). Additionally contrasting results find that women show more ambiguity aversion than men in the domain of gain but not necessarily loss (Pulford & Gill, 2014; Schubert et al., 1999), including in a competitive tournament setting (Balafoutas & Sutter, 2019). Yet a large scale study of ambiguity attitudes failed to observe any significant gender effects (Dimmock et al., 2016).

Given this variance, it remains unclear whether the decision-making processes related to ambiguous choice differ by gender. This work aims to aid in resolving these issues by taking an approach to multilevel uncertainty that starts with information processing and explicitly addresses both its risk and ambiguity components. Notably, the prior ambiguity studies are generally defined by the degree to which information is unavailable or unknown. One option for generating additional insights is to instead consider how individuals use the (partial) information that they do know. This is the perspective taken by the Pro/Con task (Peysakhovich & Karmarkar, 2016).^1^ Similar to commonly used balls-and-urns experiments, Pro/Con participants play multiple rounds of a game in which a single poker chip is drawn from a bag containing 100 chips, all of them red or blue. A red chip draw yields a monetary payout and participants indicate their estimated value for each gamble by declaring their willingness to pay (WTP) for a ticket to play the game. On each round participants are given varying levels of (partial) information (e.g., “You know at least 25 chips are red, and at least 30 chips are blue”). The number of red chips revealed represents the amount of information available that is favorable toward a winning outcome, and the blue chips revealed represent the amount of unfavorable information.

Because this design can separately vary the amount of red and blue chips revealed, it uniquely allows measurement of how each type of information relates to WTP and whether they impact it similarly. Because people are disproportionately averse to losses and negatives in general (Baumeister et al., 2001; Kahneman & Tversky, 1979; Peeters & Czapinski, 1990; Rozin & Royzman, 2001), and models of ambiguity aversion can assume pessimistic inferences (e.g., Gilboa & Schmeidler, 1989), one might predict that decision-makers would be disproportionately influenced by unfavorable information. Instead, Pro/Con results demonstrate that favorable information appears “overweighted” in WTP for ambiguous financial prospects (Buckholtz et al., 2017; Peysakhovich & Karmarkar, 2016).

Are there gender differences in the expression of this information processing asymmetry? As a preliminary step to answer this question, I reanalyzed data from Experiment 1 from Peysakhovich and Karmarkar (2016), which had a sample of 31 female and 69 male participants who played a nine-round version of Pro/Con (see Supplement A for further detail). WTP was regressed on the number of red and number of blue chips revealed, gender (female = 0, male = 1), and interactions between gender and information, with standard errors clustered at the participant level (Table 1). While there was no main effect of gender, its interaction with the amount of favorable information was significant. Specifically, increasing favorable information increased WTP for male participants more than it did for female participants, suggesting that the asymmetric impact of favorable information is amplified in men compared to women.

**Table 1:** Regression model results for analysis of factors impacting WTP in the Pro/Con task in Experiment 1 of Peysakhovich and Karmarkar (2016; Column 1), and in Pro/Con Ambiguity (Columns 2 and 3)

	WTP (2016 data)	WTP	WTP
# Red	0.0764 (0.0120)***	0.0825 (0.00301)***	0.0714 (0.00356)***
# Blue	−0.002 (0.00636)	−0.0323 (0.00225)***	−0.0273 (0.0026)***
Gender	−0.248 (1.064)		−0.106 (0.235)
# Red X gender	0.0924 (0.0248)***		0.0261 (0.00596)***
# Blue X gender	−0.00713 (0.00964)		−0.0118 (0.0046)*
Constant	4.705 (0.683)***	2.595 (0.117)***	2.64 (0.156)***
R^2^	0.1463	0.3418	0.3532
N (Participants)	900 (100)	2756 (212)	2756 (212)

Standard errors are reported in parentheses, and clustered at the participant level. ***p < 0.001; *p < 0.05

This result illustrates how this information-centered approach could offer novel insight into how gender impacts uncertain decision-making. In addition, an important challenge in qualifying gender differences in uncertainty arises from addressing and defining the relative contribution of sensitivity to risk versus sensitivity to ambiguity (Powell & Ansic, 1997; Borghans et al., 2009.) As alluded to, evaluating an ambiguous prospect effectively requires individuals make a subjective estimate of risk and then define the value of that estimate. Addressing this, an extended version of the Pro/Con task can also be used to demonstrate the impact of favorable and unfavorable information on people’s estimates of risk, and separately, their sensitivity to ambiguity (Buckholtz et al., 2017; Peysakhovich & Karmarkar, 2016). The framework for this arises from considering what information “informs” (Fig. 1). Chip color information can be used by a decision-maker as a basis for estimating the likelihood of a red chip draw (the win probability). In addition, awareness of relative ignorance and/or subjective feelings of knowledgeability are important factors in determining how ambiguity aversion is expressed and its impacts on decision-making (Fox & Tversky, 1995; Hadar et al., 2013). Regardless of whether it is favorable or unfavorable, the more chip color information people have, the less ambiguity they experience, and thus the more certain they may feel about their appraisal of the situation. Adding measures of the estimated likelihood of winning and certainty about that estimate on each round of Pro/Con can allow analysis of those constructs’ contributions to value.

**Fig. 1:**
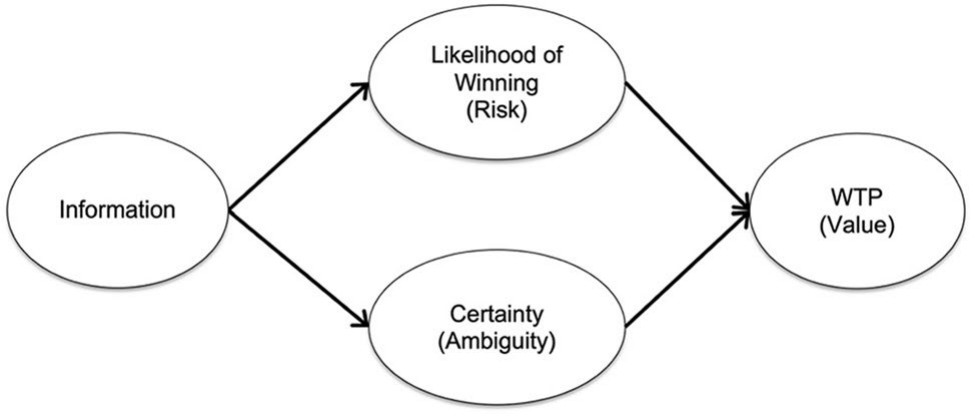
Pathways by which information influences subjective value

To pursue these questions, in Experiment 1, I conducted an incentive compatible version of the extended Pro/Con task in a sample large enough to better allow between-gender comparisons. While gender did not exert significant main effects, I find male participants use information “more optimistically” in their valuation of financial prospects than their female counterparts across the measures taken. Specifically, male participants show an amplified increase in subjective value due to increases in favorable information (consistent with the analysis of published data above), and also a diminished response to unfavorable information. Exploring the pathways illustrated in Fig. 1 reveals that these overall effects occur due to multiple points of moderation. First, there are differences in how information is used to estimate the likelihood of an outcome (risk) as well as feelings of certainty about that estimate (ambiguity). However, there are second-stage influences as well. Given a particular level of estimated likelihood, male participants assign a higher subjective value to the financial prospect. In a second experiment, I show that such gender differences in subjective value across risk levels are observed across subjectively estimated likelihoods in ambiguous prospects *and* the objectively defined likelihoods in purely risky prospects. Collectively, these findings suggest that there are gender differences in the decision processes that take place during ambiguous choice and that this is driven by multiple mechanisms across perceptions of both levels of uncertainty as well as in estimating subjective value.

## Methods

### Experiment 1: Pro/Con ambiguity

Research procedures for all experiments in this work were reviewed and approved by the UCSD Human Research Protections Program. This experiment was pre-registered on AsPredicted (https://aspredicted.org/qn2bv.pdf). Consistent with the pre-registered estimate and based on in-person recruitment availability, a sample of 220 participants (M_Age_ = 20.22, F = 126) was collected in a behavioral laboratory with a known policy preventing the use of deception. The majority of the sample (215/220) was enrolled as students at the university at the time of the experiment. Data were collected by using Qualtrics software (Qualtrics, Provo, UT) and analyzed with StataSE software (v17; Stata Corp, 2021). All participants received monetary compensation for their time. In line with the preregistered analysis plan, eight participants were excluded from the analysis for failing to answer accurately all of three attention check questions related to the experiment instructions. This resulted in a final sample of 212 participants (122 F, M_Age_ = 20.19). Participants indicated the gender they currently identified with as male, female, or other; all participants in the sample identified as male or female. Participant instructions, survey instruments, and data are available on ResearchBox (https://researchbox.org/767).

Participants provided informed consent and were endowed with 10 USD beyond their base compensation to be used in their decisions during the experiment. Instructions for the task were read aloud by an experimenter while participants followed along with their own copies. Participants engaged in 13 rounds of the Pro/Con task (e.g., the pull-a-chip game; Peysakhovich & Karmarkar, 2016). Each round reflected an independent game in which participants were asked to envision a bag containing exactly 100 poker chips, all of which were colored either red or blue. Participants were asked to indicate their willingness to pay for a “red chip ticket” using a slider from $0-$10 to play a game in which a single chip was drawn from the bag. If a red chip was drawn, participants would win $20; there was no payout if a blue chip was drawn and an implicit loss of the ticket price. On each round participants received partial information about the color contents of the bag (e.g., “You know that the bag contains at least 17 RED chips and at least 20 BLUE chips.”) Thus, the number of red chips represented the amount of favorable information the participant received, and the number of blue chips represented the amount of unfavorable information. The true underlying color composition differed for each bag. The revealed red and blue chip information was consistent with the bag’s true composition and varied between 0-50 across rounds, with no significant correlation between the numbers of red and blue chips (r = 0.144, *p* = 0.638; see also materials in ResearchBox.)

In addition to their WTP for a ticket, participants indicated their estimated likelihood that a red or a blue chip would be drawn from the bag on a bipolar 11-point scale with endpoints marked as “definitely red” to “definitely blue.” This scale was reverse coded for the reported analyses such that higher scores represented a higher estimated likelihood of drawing a red chip to facilitate its interpretation as estimated likelihood of winning. Participants also rated how certain they felt that this likelihood estimate was correct on a seven-point Likert scale labeled as “not very certain” to “extremely certain.” In addition to age and gender, participants rated the degree to which they agreed with the statement, “Most people can be trusted.” Trust ratings did not differ by gender (*p* = 0.607) and were not analyzed further.

The study was incentive compatible, with one decision randomly chosen to count “for real” and played once all task measures had been completed. On each round, WTP was elicited using a version of the Becker-DeGroot-Marschak procedure (Becker et al., 1964) explained in detail to participants during the instruction phase. No feedback on the outcome of any round was provided during the main task. When the round to be played was chosen, the experimenter used a random number generator (https://www.random.org/integers/) to set a ticket price between 1 and 10 USD, inclusive. If participants had indicated a WTP higher than this price, they paid the ticket price and the round’s game was played out. If they indicated a WTP lower than the price, they retained their endowment. The game was played by generating a random number between 1 and 100, inclusive. If the number generated was less than or equal to the *true* number of red chips contained in the bag, this signaled that a red chip was “drawn,” resulting in payouts of 20 USD for participants who had purchased tickets.

### Experiment 2: Pro/Con ambiguity vs. Pro/Con risk

Research procedures were reviewed and approved by the UCSD Human Research Protections Program. An experiment designed to allow comparison between subjective and objective risk versions of Pro/Con was preregistered on AsPredicted (https://aspredicted.org/tk8sq.pdf). The experiment was conducted with a distinct sample from Experiment 1, comprised of participants from Amazon Mechanical Turk (n = 603; M_Age_ = 40.61; 267 F, 9 NB) via the Cloud Research platform. Data were collected by using Qualtrics software (Qualtrics, Provo, UT) and analyzed with StataSE software (v17; Stata Corp, 2021). Participants indicated the gender they currently identified with as male, female, or other. In line with the preregistered analysis plan, 35 participants who were unable to correctly answer three comprehension questions related to the task instructions and nine participants who did not identify as male or female were excluded from analysis, resulting in a sample of 559 individuals (M_Age_ = 40.82, F = 254). Survey instruments and data are available on ResearchBox (https://researchbox.org/767).

All participants engaged in rounds of hypothetical Pro/Con-style games. As in Experiment 1, each round reflected an independent game in which participants were asked to envision a bag containing exactly 100 poker chips, all of which were colored either red or blue. A red chip draw was described as resulting in a payout of 50 USD; there was no payout if a blue chip was drawn. Participants indicated their WTP for a “red chip ticket” on a slider from 0 to 40 USD to play a game in which a single chip was drawn from the bag. Unlike Experiment 1, there was no BDM instruction or procedures associated with this question, nor did participants have reason to expect the games would be played out.

The sample was randomly assigned across two distinct versions of the experiment. In the Ambiguity Pro/Con, participants (n = 274, 131 F) viewed information across 13 rounds as in Experiment 1. They indicated their WTP and their estimated likelihood of drawing a blue or red chip on the same scale as Experiment 1. This scale was again reverse coded for the reported analyses such that higher scores represented a higher estimated likelihood of drawing a red chip to facilitate its interpretation as estimated likelihood of winning. In the Risk Pro/Con, participants (n = 285, 123 F) received complete information about the chip color contents of the bag on each round (e.g., “You know that the bag in front of you contains exactly 17 RED chips and 83 BLUE chips”). Red chip information thus indicated the objective probability of winning and varied between 3% and 96% (inclusive) across 12 rounds.^2^

All participants indicated age and gender as in Experiment 1. In addition, participants indicated approximate formal educational experience across five levels: 1 = less than high school; 2 = high school graduate; 3 = some college; 4 = bachelors or 4 year degree; 5 = professional degree or doctorate. They also indicated approximate household income across 7 levels starting with 1 = $0-$24,999 up to 7 = $150,000 in $25,000 increments with an additional option of 8 = decline to state.

## Results

### Experiment 1: Information processing in ambiguous decisions

As preregistered, to establish replication of prior findings, willingness to pay (WTP) was regressed on the number of red chips revealed (favorable information) and the number of blue chips revealed (unfavorable information), with standard errors clustered at the participant level. Favorable information showed a significant positive impact on WTP, while unfavorable information had a significant negative impact. In addition, favorable information had more influence; the magnitude (absolute value) of the coefficient on the favorable information was larger than the coefficient on the unfavorable information (test for equality of coefficients; F(1,211) = 340.02, *p* < 0.001). Collectively, the findings from this data replicate the patterns observed in prior instances of the Pro/Con task (Peysakhovich & Karmarkar, 2016; Buckholtz et al., 2017; see Supplement B for the full replication analysis as preregistered).

To examine the potential for gender differences, WTP was regressed on the revealed red and revealed blue information with a categorical gender variable (0 = female, 1 = male), and terms for their respective interactions (Table 1). As observed in the analysis of the Peysakhovich and Karmarkar (2016) data, there was no significant main effect of gender, indicating that in this setting, male and female participants had similar WTP overall. However, the interactions between gender and information were *both* significant. Specifically, as favorable information increased, it became more impactful for men than women. Paralleling this, as unfavorable information increased, it had significantly less impact on WTP for men.

The complementary nature of the significant favorable and unfavorable interactions predicts that they should amplify the difference between male and female participants’ WTP as the total amount of information (number of red chips + blue chips) increases. To test this, extending beyond the preregistered analyses, WTP was regressed on the total information, gender and their interaction, with standard errors clustered at the participant level. There was a significant effect of total information (B = 0.0237, SE = 0.00177, *p* < 0.001, 95% CI [0.0202, 0.0271]), showing that value for the gamble increased as information increased, regardless of its valence. While there was no significant effect of gender alone (B = −0.137, SE = 0.236, *p* = 0.561, 95% CI [−0.601, 327]), there was a significant interaction of information and gender (B = 0.00779, SE = 0.00277, *p* < 0.01, 95% CI [0.0023, 0.0132]). In service of interpreting this, it is useful to consider that increases in the amount of information reduce the level of ambiguity, such that full information about the color contents of the bag creates a purely risk-based prospect (e.g., 50 red chips/50 blue chips). Thus, as the decisions became more risk-like, male participants demonstrated a higher WTP than their female counterparts.

As discussed, valuations of ambiguous prospects rely both on the estimated likelihood of winning and the relative (un)certainty the individual feels about those estimates (Figure 1). These findings raise the question of whether the information processing differences observed in Table 1 exert their impact on value via one or both of these pathways. In addition, since significant differences in WTP occur when the decisions are the most risk-like and ambiguity is minimal, it also can be asked whether estimated likelihood itself has higher influence on subjective value for male compared with female participants. I examine each question below.

### Experiment 1: Valenced information use in estimated risk and certainty

On each round of Pro/Con, participants estimated the likelihood that a red (versus a blue) chip would be drawn if the game was played for the bag. Rationally, increasing information about the number of red chips should increase the perceived likelihood of drawing a red chip (and thus the probability of winning), with the reverse true for blue chips. This accurately describes the results of regressing the estimated likelihood of a red chip draw on the number of red and blue chips, gender, and the interaction of gender and each form of information (Table 2, column 1). Examining the interaction of information and gender reveals that valenced information impacts perceived risk more positively and less negatively for male individuals. Specifically, increases in the number of red chips increases the estimated likelihood of winning more for men, and increases in the number of blue chips decreases the estimated likelihood of winning less for men (Table 2, column 1). This yields a net “optimism bias” in subjective risk for male, compared with female, participants.

**Table 2:** Regression model results examining factors impacting estimated risk (Column 1) feelings of certainty (Column 2) and WTP (Last Column) in the Pro/Con ambiguity task

	Likelihood red (Win)	Certainty		WTP
# Red	0.0667 (0.00342)***	0.0253 (0.00196)***	Likelihood red	0.594 (0.0379)***
# Blue	−0.0583 (0.00323)***	0.0249 (0.00203)***	Certainty	0.233 (0.0622)***
Gender	−0.0866 (0.118)	0.0603 (0.248)	Gender	−0.780 (0.505)
# Red X gender	0.0156 (0.00561)***	0.00225 (0.00309)	Likelihood X gender	0.130 (0.055)*
# Blue X gender	−0.0134 (0.00507)***	0.00799 (0.00324)*	Certainty X gender	0.0445 (0.0819)
Constant	5.688 (0.0753)***	2.753 (0.156)***	Constant	−0.693 (0.356) ^†^
R^2^	0.546	0.163	R^2^	0.341
N (212 clusters)	2756	2756	N (212 clusters)	2756

Standard errors are reported in parentheses, and clustered at the participant level

***p < 0.001; *p < 0.05; ^†^*p* = 0.053.

The complementary regression of the certainty measure on revealed amounts of favorable and unfavorable information demonstrates that both increase felt certainty (Table 2, column 2). This is consistent with the idea that any increase in information, regardless of valence, recognizably increases knowledge and decreases the degree of ambiguity for the participants. Interestingly, however, while there is no significant interaction of gender and favorable information, increases in unfavorable information make male participants feel more certain about their risk estimates than women. Considered from the perspective of information processing, the likelihood and certainty data suggest that male participants’ perceptions of both layers of uncertainty are more sensitive to changes in available information.

### Experiment 1: Contributions of subjective risk and certainty to value

In addition to the question of how information impacts subjective perceptions of risk and feelings of certainty, the Pro/Con framework raises a question of whether male participants might subsequently also weight those dimensions differently when determining the value of the gambles in each round. To test this, in line with the pre-registered analyses, WTP was regressed on the likelihood of winning, certainty, gender, and the interaction of gender with each rating type with standard errors clustered at the participant level. As demonstrated in Table 2, column 3, increases in the estimated likelihood of winning and felt certainty both significantly increase WTP, confirming the relevance of both pathways in the decision process.^3^ In addition, a significant interaction of likelihood and gender demonstrates that male participants increased their WTP more as their likelihood of winning increased. The interaction of certainty and gender was not similarly significant. Overall, analyses of Experiment 1 find that gender differences in information processing under ambiguity are present both in terms of risk and ambiguity components but are particularly pronounced in relation to risk as measured as the estimated likelihood of winning the gamble.

### Experiment 2: Comparing gender differences in value of increasing subjective and objective risk

Since Experiment 1 did not include trials with varying levels of objective risk, it is unclear whether the interaction relationship between gender and risk level on value is unique to subjectively estimated risk (e.g., risk estimated under ambiguity.) To investigate this, I conducted a second experiment allowing comparisons of this relationship between the ambiguity-based Pro/Con task to a “risk only” version of Pro/Con in which participants are given full information about the color contents of bags with varying objective probabilities of winning. Specifically, in the Risk version, participants were given the exact number of both red and blue chips in the bag, while in the Ambiguity version, the partial information presented was identical to the values used in Experiment 1. In both these hypothetical-stakes experiments, drawing a red chip would result in a win of $50 and a blue chip would result in $0, with the implicit loss of the price of the ticket.

**Fig. 2:**
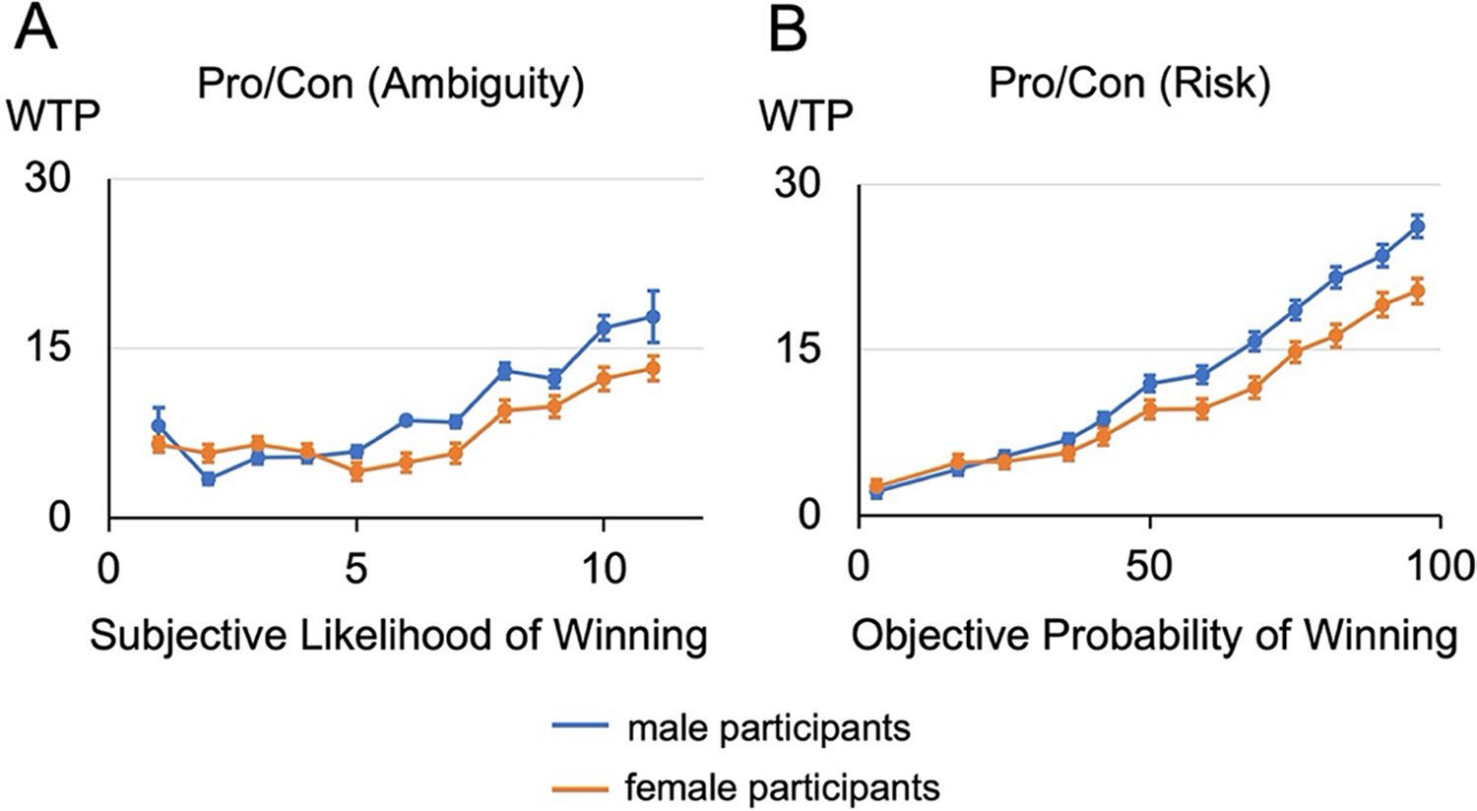
Relationship between WTP and risk perceptions in Experiment 2. **(A)** WTP as a function of estimated likelihood of drawing a red chip in the Pro/Con ambiguity task. **(B)** WTP as a function of probability of drawing a red chip in the risk Pro/Con task

As preregistered, for the Ambiguity version, WTP was regressed on the subjective likelihood of winning, a categorical variable for gender (0 = female, 1 = male) and their interaction, with standard errors clustered at the participant level (R^2^ = 0.102, *p* < 0.001). Replicating the pattern of incentive compatible findings in Experiment 1, there was a significant effect of likelihood on willingness to pay (B = 0.668, SE = 0.209, *p* < 0.01, 95% CI = [0.256, 1.081]). The coefficient on gender was not significant (*p* = 0.469). However, the significant interaction of gender and subjective likelihood replicated here as well (B = 0.626, SE = 0.253, *p* = 0.014, 95% CI = 0.127, 1.124]; Fig. 2A).

For the Risk version, in line with the preregistration, WTP was regressed on the objective probability of winning (signified by number of red chips), a categorical variable for gender (0 = female, 1 = male), and their interaction, with standard errors clustered at the participant level (R^2^ = 0.326, *p* < 0.001.) As would be expected, there was a significant effect of win probability on WTP (B = 0.192, SE = 0.0135, *p* < 0.001, 95% CI = [0.166, 0.219]). While prior research might have generated the prediction that women would consistently have lower valuations for risky prospects than men, there was no significant main effect of gender (*p* = 0.209). However, paralleling the ambiguity analyses, there was a significant interaction such that male participants increased their WTP proportionally more than female participants as the likelihood of winning increased (B = 0.0720, SE = 0.0192, *p* < 0.001, 95% CI = [0.0342, 0.110]; Figure 2B). This pattern of effects for the Risk Pro/Con task was further replicated in an independent experiment conducted on separate participant sample (see Supplement D.)

While the differences in the dependent variable offer challenges for a direct statistical comparison of these results, a similar interaction is observed between gender and risk for WTP, as illustrated across the panels in Fig. 2. Extending beyond the preregistered analyses, regressions controlling for age, education, and income did not alter the significance of these interactions (see Supplement C for all models’ full results.) This suggests that regardless of whether the risk is exogenously presented objectively or endogenously estimated subjectively, male participants significantly increase their WTP more than female participants as the perceived likelihood of winning increases. Put differently, while estimation of value under ambiguity is almost certainly multiply determined, these results also show that this gender sensitive mechanism of risk processing may be conserved in situations with incomplete information processing.

## Discussion

These experiments find gender differences in valuation of ambiguous financial prospects arising from a more “optimistic” use of information for male compared to female participants. As favorable and unfavorable information accrues (reducing ambiguity) male participants interpret it in ways that more support an increased likelihood of a winning outcome. In addition, they give unfavorable information additional weight in reducing feelings of uncertainty. This positively skewed information processing is further amplified across the risk pathway in Figure 1—as the estimated likelihood of winning increases, the size of the gender difference in valuation further increases. The net impact of these individual risk and certainty factors is a degree-dependent form of relative optimism. There is little gender difference in value when there is “complete” ambiguity of the kind described by the Ellsberg paradox (1961), but the gap between genders increases as increases in information decreases ambiguity and is maximal when ambiguity goes to zero (e.g., in risk). Another way of interpreting these data is that male participants are more influenced by changes in the amount of available information than female ones were. That is, if willingness to pay for a purely risky 50/50 gamble is used as a baseline condition, female participants deviate less from that baseline as the amount of information decreases (and ambiguity increases).

As noted, the results show significant gender differences in valuation are driven by risk-related mechanisms, consistent with prior findings that men assign a higher value to risky financial prospects (Charness & Gneezy, 2012). However, the present results are not due to a main effect in which males show consistently higher WTP. The task used for this research allows a nuanced set of insights by varying both the (implied) risk probabilities as well as the degree of ambiguity in the financial prospects presented in each round. Similar to their sensitivity to the degree of ambiguity, male participants in the present experiments were more sensitive to the degree of risk, showing a higher increase in value as the probability of winning increased. Furthermore, in Experiment 2, this pattern of risk valuation was observed across ambiguous decisions (in which likelihood was subjectively estimated) and risky decisions (in which likelihood was objectively known), raising the possibility that those mechanisms are conserved across settings.

These behavioral findings offer a meaningful step for advancing a neuroeconomic understanding of uncertain decision-making as it occurs in real world contexts, including differential expression by gender. Using neuroimaging techniques to study gender differences can have practical challenges as they entail between-subject rather than within-subject comparisons. This requires larger sample sizes, thus enhancing the need to select a sufficiently comprehensive task that has been demonstrated to show such effects robustly. The identification of multistage mechanisms in this work offers an opportunity to make hypotheses based on distinct neural mechanisms associated with information type (favorable vs. unfavorable) in addition to the more obvious tests of moderating risk and ambiguity parameters.

In the expansive literature on the neural mechanisms of uncertain decision-making, studies on gender differences in brain activity have focused on risk (Lighthall et al., 2012; Zhou et al., 2014; Zhang et al., 2017). One commonly used paradigm for this is the Balloon Analog Risk Task (BART; Lejuez et al., 2002), in which participants make sequential decisions that trade off the opportunity to gain money by adding more inflation to a balloon versus an increasing risk of losing all of their funds if choosing to inflate the balloon more causes it to pop. Consistent with fMRI findings on other risk tasks (Preuschoff et al., 2008), increasing levels of risk (as the balloon gets larger) in the BART have been shown to correlate with activity in areas, such as anterior insula (Rao et al., 2008; Korucuoglu et al., 2020). Comparisons of neural activity between genders, however, have observed differences primarily correlated with decision-making and outcomes, with female participants showing increased activity in multiple ROIs, including insula and lateral orbitofrontal cortex during loss (pop) outcomes (Korucuoglu et al., 2020).

While this task is labeled in terms of risk, it can be argued it also involves ambiguity. Each increase in inflation offers partial information about the likelihood of the balloon popping, but the specific probability that the next decision will cause a pop is not necessarily presented to participants (Lejuez et al., 2002). As a mix of risk and ambiguity it offers potentially interesting comparisons and predictions for employing Pro/Con as an fMRI task. For example, an fMRI BART study found significant activity in dorsal lateral prefrontal cortex (dlPFC) associated with deciding whether or not to inflate the balloon as well as the decision outcome (Rao et al., 2008). A functional near-infrared spectroscopy replication of this work found that dlPFC activity differed by gender -- female participants showed higher increases in dlPFC activity during loss decisions (Cazzell et al., 2012). This raises the question of how dlPFC activity might vary by gender in response to “aversive” ambiguous stimuli implying a high loss probability in contrast to those that are aversive due to high levels of ambiguity. In addition, the conservation of gender moderation across subjective and objective likelihoods in Experiment 2 suggests that the networks observed in the BART research would be appropriate as a priori hypothesized regions of interest (ROIs) for gender differences.

Similar regions of interest would arise from multiple studies finding that the circuitry involved in processing ambiguity and risk is differentiable (Blankenstein et al., 2017; Blankenstein et al., 2018; Hsu et al., 2005; Huettel et al., 2006; Levy et al., 2010; Wu et al., 2021). Integrating these findings suggests that it would be possible to test the multiple processes that can be moderated by gender illustrated in Fig. 1. It also raises interesting questions about whether there would be observable differences in neural patterns of activity between genders for risk versus ambiguity.

Relatedly, work demonstrating that the subjective value of risky and ambiguous prospects have similar neural representation in reward-related circuitry (Levy et al., 2010) offers the straightforward prediction that gender differences should be observed in reward networks. However, using an information-centered task, such as Pro/Con, also would offer the possibility of observing whether favorable and unfavorable information engages the same subjective value circuitry and whether it does so similarly by valence. Overall, there are meaningful opportunities for not only resolving apparent conflicts in the literature but also for theory-building based on neural correlates of each stage of this task.

Prior work has shown that there are gender differences in optimism or positive expectations about future outcomes (Carver et al., 2010), distinct from risk preferences. For example, Jacobsen et al. (2014) found that increased optimism could account for men’s higher investments in risky assets (e.g., stocks). Similarly, Bjuggren and Elert (2019) found that Swedish men expressed more optimism about their country’s economic future than women. As another example in a different domain, it was found that men were more optimistic than women about marriage and divorce outcomes (Lin & Raghubir, 2005). As shown in the first column of Table 2, male participants in the Pro/Con interpreted both favorable and unfavorable information in a way that increased their estimated likelihood of a good outcome compared with female participants. This may offer an information-processing based mechanism underlying optimistic expectations worth exploring in future work.

It is important to recognize the constraints of these experiments and the limitations they entail. First, gender identification was elicited via self-report and cannot be directly linked to sex or genetic mechanisms that might drive differences in sensitivity to uncertainty. As has been discussed in the context of gender and risk (Booth & Nolen, 2012), the causes of the scope of the differences found in gender and uncertainty are unlikely to be clearly attributable to nature or nurture. Second, other socioeconomic, demographic, cultural, or psychological factors may exert their own influence on evaluations of uncertainty and can be correlated with gender (D’Acunto et al., 2021). The data in this paper do demonstrate convergent effects related to favorable information across three sample populations (at different points in time). In addition, controlling for factors, such as age, household income, and relative level of education, did not impact the pattern of findings (Supplement C). Furthermore, some SES individual differences themselves arise from, or are impacted by, gender. However, the range of possible influences is quite large, and there may be additional moderating variables of interest that were relatively homogenous among the experimental samples (such as access to digital technologies, for example). Thus, it would be ideal to pursue large-scale replications of these effects in samples that include panels of demographic variables of interest to understand the robustness of the gender-specific effects.

There also is the consideration that these findings are specifically demonstrated using only the Pro/Con task. This raises several opportunities for expanding the scope of the investigation to other tasks. For example, as suggested by the variety of ways to elicit risk preferences (Charness et al., 2013), uncertainty preferences in tasks with similar designs to Pro/Con can be measured with a range of dependent variables, such as multiple price lists, decisions between a gamble and a certainty equivalent, or choices between ambiguous and risky decisions. In addition, the question of gender differences could be tested in tasks that feature qualitatively different forms of uncertainty. For example, these versions of Pro/Con depended on a pull-a-chip game involving aleatory uncertainty around a currently undetermined (inherently stochastic) outcome but could be measured in tasks involving epistemic uncertainty related to awareness of ignorance (see also Fox & Ülkümen, 2011). Preliminary findings from an exploratory study using an epistemic version of the Pro/Con task show that the interaction between gender and favorable as well as unfavorable information in determining subjective value can hold in this context (reported in Supplement E), suggesting promising results for such research. Advancing beyond behavior to add neuroimaging, as noted, comparison with the circuitry that show different patterns of activity by gender during the BART would aid in determining whether gender is operating on a conserved set of mechanisms or whether there are additional task-specific constructs that are moderated.

Taking a broader view of this point, research examining gender differences in risk also has shown that such differences are strongly dependent on the specifics of the decision setting and that they may not be found across naturalistic contexts (Schubert et al., 1999). Of note, the primary Pro/Con task was conducted with real monetary consequences, suggesting that it may at least extend to other forms of financial behavior. Furthermore, differences in (lab-measured) ambiguity aversion have been shown to correlate with other forms of decision-making (Muthukrishnan et al., 2009), as well as facets of psychiatric diagnoses (Buckholtz et al., 2017; Ruderman et al., 2016), suggesting their usefulness in understanding a wider range of behavior. However, uncertainty preferences can indeed be domain-specific (Blais & Weber, 2006), and further examination would help to define the appropriate range of generalization possible from these effects. In addition, the present findings illustrate the importance of the amount and nature of available information in a situation and how this can moderate the magnitude of gender differences in uncertainty. Thus, it may be useful to measure or manipulate degree of domain-relevant perceived expertise in future work.

Overall, I demonstrate that male individuals show optimistic interpretations of both favorable and unfavorable information during ambiguous decision-making compared to female counterparts. In addition, these differences are significantly dependent on risk-related processing, consistent with prior research on gender differences in risk preferences. Individual differences in sensitivity to both risk and ambiguity have the potential to shape the demographics of who engage in different types of market activity (Balafoutas & Sutter, 2019). Thus, in addition to an enhanced psychological understanding of behavior under uncertainty, and a pathway forward for neuroscientific investigation, these results may offer insights for applications such as developing policy and support for individual-level financial welfare.

## Supplementary Information

ESM 1 (DOCX 29.4 KB)

## Data Availability

Preregistration documents, survey instruments, participant instructions, data, and additional supporting material are available at the following Research Box link: https://researchbox.org/767

## Footnotes

1 The task is referred to as the pull-a-chip game in the original publication.

2 Due to an error in the survey, no information was presented during one of the 13 rounds. This round was thus excluded from analysis.

3 The certainty measure asks participants to indicate how certain they were that their likelihood estimate was correct. To support the use of both variables in the same model, the certainty responses were separately regressed on the likelihood measure (with standard errors clustered by subject). The coefficient on likelihood was not significant (B = −0.019, SE = 0.012, *p* = 0.12). However, it remains possible that there are more complex relationships between certainty and likelihood.
