# Supplementary material for: Gender differences in “optimistic” information processing in uncertain decisions
Source: Cogn Affect Behav Neurosci. 2023 Feb 23;23(3):827–37. doi: 10.3758/s13415-023-01075-7 (PMC10390607; doi:10.3758/s13415-023-01075-7)
Supplement: Supplementary file 1 — (DOCX 29.4 KB) [file 13415_2023_1075_MOESM1_ESM.docx]

**Supplementary Materials for “Gender Differences in ‘Optimistic’ Information Processing in Uncertain Decisions”**

**Supplement A:** Gender Differences in Experiment 1 from Peysakhovich and Karmarkar (2016)

Data reported in Experiment 1 of Peysakhovich and Karmarkar (2016) was (re-)analyzed

as a preliminary investigation of gender differences in the use of information in ambiguous decision-making.

The data reflected 117 individuals who had participated in a 9-round version of the Pro/Con task via the Amazon Mechanical Turk platform. The experiment had included three comprehension questions. Following the procedures in the original publication, participants who failed to answer any of these three questions were excluded from the analysis, resulting in a sample of 100 individuals (M_Age_=32.81, 39 F). Data was analyzed using StataSE software (v17; Stata Corp 2021.) As a brief summary of the task, participants in this hypothetical stakes experiment had been informed that drawing a red chip from a bag of 100 red and blue chips would result in winning $50, and that pulling a blue chip would result in winning nothing. Partial information about the color contents of the bag had been revealed such that participants learned there were at least *R* red chips and at least *B* blue chips, where *R* and *B* were varied parametrically from 0 to 50 (inclusive) in 25 chip increments. Participants had indicated their willingness to pay (WTP) for each gamble on a slider with a scale that ranged from $0 to $35.

WTP was regressed on the number of red chips revealed, the number of blue chips revealed, gender, and interactions between gender and valenced information, with standard errors clustered at the participant level. Table A1, column 1 shows that the number of red chips (favorable information) is a significant predictor of WTP, but also that there is a significant interaction of this variable with gender. Specifically, as the amount of favorable information increases, male participants increase their WTP more than female ones do. Thus, male participants show an exaggerated form of the information processing asymmetry observed in the original publication. As a secondary control, Table A1, column 2 shows that this result is similarly present if an interaction term for the number of red and number of blue chips is also included in the model.

**Table A1**

|  | **WTP** | **WTP** |
| --- | --- | --- |
| # red | .0764  (.0120)*** | .894  (.045)*** |
| # blue | -.002  (.00636) | .011  (.0103) |
| gender | -.248  (1.064) | -.249  (1.065) |
| # red X gender | .0924  (.0248)*** | .0924  (.0241)*** |
| # blue X gender | -.00713  (.00964) | -.00713  (.00964) |
| # red X # blue |  | -.00052  (.00272) |
| constant | 4.705  (.683)*** | 4.38  (.661)*** |
|  |  |  |
| R^2^ | .1463 | .1470 |
| N  (Participants) | 900  (100) | 900  (100) |

****p<.001*

**Supplement B:** Experiment 1 Replication Analyses for Pro/Con (Ambiguity)

The incentivized Pro/Con Ambiguity task in the main paper employs the same overall design as the “pull-a-chip” task in Experiment 5 of Peysakhovich and Karmarkar (2016). In addition, as shown in the main paper (Table 1, Column 2), the data replicates the primary finding that favorable information impacts willingness to pay (WTP) more than unfavorable information does. However, this research employs a significantly larger sample, a smaller endowment and stakes, fewer rounds, and different specific chip compositions for the bags in each round. Thus, as pre-registered (https://aspredicted.org/HY1_F5D) it is useful to confirm that the results from this experiment replicate the full qualitative pattern of findings observed in the earlier publication. This effectively involves conducting the same regression analyses as those in the main paper without the gender variable.

Overall, the data collected in this paper replicates the findings in Peysakhovich and Karmarkar (2016). All regressions reflect 2756 observations, with standard errors clustered at the participant level (212 clusters). Table B1 Column 1 shows that the estimated likelihood of winning is significantly increased by increases in the number of red chips (favorable information) and similarly decreased by increases in the number of blue chips (unfavorable information.) In Table B1 Column 2, regressing certainty ratings on the two types of information shows that both significantly *increase* felt certainty, with comparable magnitudes. The last column of Table B1

reflects how the risk-related (likelihood) and ambiguity-related certainty) components inform WTP. Specifically, increases in both likelihood and certainty significantly increase WTP.

**Table B1**

|  | **Likelihood Win** | **Certainty** |  |  | **WTP** |
| --- | --- | --- | --- | --- | --- |
| # red | .0734  (.00278)*** | .0263  (.00152)*** |  | Likelihood Win | .657  (.0283)*** |
| # blue | -.0640  (.00253)*** | .0283  (.00161)*** |  | Certainty | .256  (.0422)*** |
| constant | 5.652  (.058)*** | 2.779  (.122)*** |  | constant | -1.078  (.261)*** |
|  |  |  |  |  |  |
| R^2^ | .540*** | .153*** |  | R^2^ | .336*** |

****p<.001*

**Supplement C:** Experiment 2 Extended Regression Models Including Demographic Factors

Participants in Experiment 2 provided demographic information including age, education attained, and household income in addition to gender identification. Regression of the Ambiguity and Risk Pro/Con data from this experiment demonstrates that the interaction between gender and (subjective or objective) likelihood remains significant when controlling for these factors. Note that the number of observations is smaller in the models that include the control variables, as some participants chose not to indicate their level of household income.

**Table C1**

|  | **WTP Ambiguity** | **WTP Ambiguity** |  | **WTP Risk** | **WTP Risk** |
| --- | --- | --- | --- | --- | --- |
| Likelihood (Risk) | .669  (.00278)** | .679  (.212)** |  | .192  (.0135)*** | .192  (.0138)*** |
| Gender | -1.227  (1.691) | -1.187  (1.705) |  | -1.272  (1.010) | -.1.510  (1.056) |
| RiskXGender | .626  (.253)* | .611  (.256)* |  | .0720  (.0192)*** | .0740  (.0196)*** |
| Age |  | -.0256  (.0150) |  |  | -.121  (.0361)** |
| Education |  | .221  (.430) |  |  | .715  (.523) |
| Income |  | .107  (.249) |  |  | -.240  (.284) |
| Constant | 2.334  (1.418) | 2.252  (1.841) |  | .214  (.779) | 3.453  (2.347) |
|  |  |  |  |  |  |
| R^2^ | .110*** | .108*** |  | .326*** | .341*** |
| N  (Participants) | 3549  (273) | 3471  (267) |  | 3705  (285) | 3348  (279) |

****p<.001, **p<.01, *p<.05; standard errors clustered at the participant level*

**Supplement D**: Replication of the Risk Pro/Con Findings

This experiment replicates the Risk-only findings component of Experiment 2 from the main manuscript. Research procedures were reviewed and approved by the UCSD Human Research Protections Program. A risk-only version of Pro/Con with hypothetical stakes was pre-registered on AsPredicted (https://aspredicted.org/72nb6.pdf). The experiment was conducted with participants from Amazon Mechanical Turk (n= 301; M_Age_=40.44; 136 F, 3NB) via the Cloud Research platform. Data was collected using Qualtrics software (Qualtrics, Provo, UT) and analyzed with StataSE software (v17; Stata Corp 2021). Participants indicated the gender they currently identified with as male, female, or other. In line with the pre-registered analysis plan, 27 participants who were unable to correctly answer three comprehension questions related to the task instructions and three participants who did not identify as male or female were excluded from analysis, resulting in a sample of 271 individuals (M_Age_=40.44, F = 124). Survey instruments and data are available on ResearchBox (https://researchbox.org/767).

Participants engaged in 11 rounds of hypothetical risk-based games. As in Experiment 2, each round reflected an independent game in which participants were asked to envision a bag containing exactly 100 poker chips, all of which were colored either red or blue. A red chip draw was described as resulting in a payout of $50; there was no payout if a blue chip was drawn. Participants indicated their WTP for a “red chip ticket” on a slider from $0 to $40 to play a game in which a single chip was drawn from the bag. On each round participants received complete information about the chip color contents of the bag (e.g., “You know that the bag in front of you contains exactly 17 RED chips and 83 BLUE chips.”) Red chip information thus indicated the objective probability of winning and varied between 10% and 90% (inclusive) across rounds. Participants indicated their age and gender after completing the task.

As pre-registered, WTP was regressed on the probability of winning (signified by number of red chips), a categorical variable for gender (0 = female, 1 = male), and their interaction, with standard errors clustered at the participant level (R^2^ = .352, p<.001.) There was a significant effect of win probability on WTP (B = .171, SE =.013, p<.001, 95% CI = [.144 .197]), but no significant main effect of gender (p = .226). In addition, there was a significant interaction such that male participants increased their WTP proportionally more than female participants as the likelihood of winning increased (B = .110, SE = .020, p<.001, 95% CI = [.0714 .149]). These results show an identical pattern to that observed for Experiment 2, replicating those findings in an independent sample.

**Supplement E**: Gender Differences Replicate in Pro/Con “Majority” Task

The “pull-a-chip” design of Experiments 1 and 2 reflect a form of aleatory uncertainty, arising from fundamentally random future events. In this experiment, I explore whether there are gender differences in how information is used in a “majority” version of the Pro/Con task (Peysakhovich and Karmarkar, 2016) involving epistemic uncertainty, in which uncertainty arises from ignorance about a fundamentally knowable (or already determined) outcome. In each round of the majority task, participants asked to imagine that in each round there are exactly 101 poker chips in a bag, all of which are colored red or blue. If the round is played, the contents of the bag are revealed. If the majority of the chips in the bag (51 or more) is red, they win $50, and if it is blue, they win nothing. As in the other versions of Pro/Con, participants are given partial information about the color contents of the bag on each round.

Research procedures were reviewed and approved by the UCSD Human Research Protections Program. The experiment was conducted with participants from Amazon Mechanical Turk (n= 151; M_Age_=31.78; 50 F, 1NB) via the Cloud Research platform. Data was collected using Qualtrics software (Qualtrics, Provo, UT) and analyzed with StataSE software (v17; Stata Corp 2021). Participants indicated the gender they currently identified with as male, female, or other. Similar to the exclusion criteria in Experiments 1 and 2, 49 participants who were unable to correctly answer three comprehension questions related to the task instructions and one participant who did not identify as male or female were excluded from analysis, resulting in a sample of 101 individuals (M_Age_=32.78, F =33). Survey instruments and data are available on ResearchBox (https://researchbox.org/767).

Participants engaged in 15 rounds of a hypothetical stakes majority game and indicated their willingness to pay (WTP) for a ticket to play the game on a scale from $0 to $35 on each round. They then reported their age and gender and rated their agreement with the statement “Most people can be trusted.” Participants were provided an opportunity to leave comments or feedback at the end of the survey; the majority of individuals left this space blank.

To analyze how the use of favorable and unfavorable might differ by gender, WTP was regressed on the number of red chips revealed, the number of blue chips revealed, gender (0 = female, 1 = male), and the interaction of each type of information with gender. The results are presented in Table E1. Replicating the pattern of results seen in Experiment 1, there was a significant effect of both favorable (red) and unfavorable (blue) information on subjective value as measured by WTP. While there was no main effect of gender, its interactions with valenced information were also significant. As observed in Experiment 1, male participants increased their subjective value more for increases in favorable information, and showed less of a decrease in value for unfavorable information. This exploratory study suggests that the influence of gender on information processing may generalize across aleatory and epistemic forms of ambiguity.

**Table E1**

|  | **WTP** |
| --- | --- |
| # red | .143  (.0252)*** |
| # blue | -.083  (.0173)*** |
| gender | 1.451  (1.573) |
| # red X gender | .0751  (.0334)* |
| # blue X gender | -.0614  (.0242)* |
| constant | 6.560  (1.342)*** |
|  |  |
| R2 | .1690 |
| N  (Participants) | 1515  (101) |

**p<.05, ***p<.001, standard errors clustered at the participant level*
